# Circulatory Assistance: the Challenges of Technological Incorporation in Brazil

**DOI:** 10.5935/1678-9741.20150091

**Published:** 2015

**Authors:** Helmgton Souza

**Affiliations:** UNIFESP-EPM, Coordinator of the Advanced Heart Failure and Circulatory Assistance Center of Hospital Brasília and Hospital Coração do Brasil - Distrito Federal, Program Director of 456th ECMO Center. Brasília, DF, Brazil.

Dear Editor,

Unfortunately, we have always recognized that technological advances incorporated into the clinical practice of the Brazilian medicine occur with some immense delay behind the so-called first world countries. There are several reasons for that, ranging from the bureaucracy of the registration and import processes to the issues related to exchange differences - leveraged in recent months, passing through voracious federal, state and local taxation, as well as the recurring economic limitation of health funding institutions, in other words, the National Health System and health insurances companies, besides the natural learning process of professionals, usually physicians, who use these technologies in the daily treatment. Inevitably, we are always lagging behind of what happens in Europe and the United States. Worse than that is to see that our neighbors, with much weaker economies than ours, can incorporate these technologies with greater ease, making us reflect: Why insist on this archaic and retrograde model?

There was a panel with the same title as this article on October 23, 2015, promoted by Hospital Brasilia, Distrito Federal, ending the 1^st^ Advanced Heart Failure and Circulatory Assistance Symposium. Hospitals, health insurance company managers, industry representatives of hospital medical equipment, government, the Federal Council of Medicine, the Judiciary, and of course, physicians who daily face the dilemma of knowing that they can always offer a little more, in light of what is already in operation in the world, but are hampered by bureaucracy, budget constraints, and even a certain amount of ignorance and inefficiency.

**Fig. 1:**
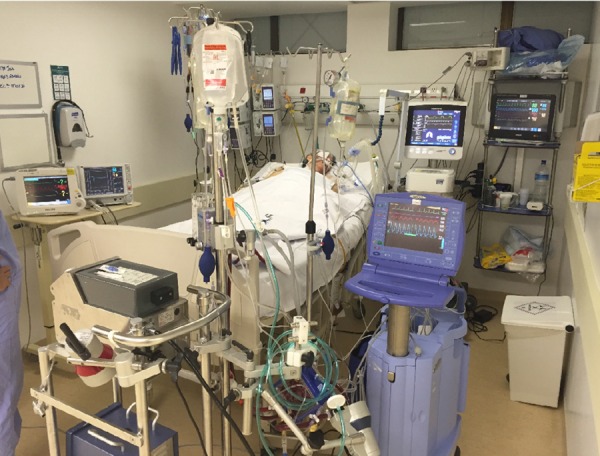
Patient in circulatory support (ECMO) - Hospital Brasília, Federal District, Brazil.

The debate could be directed to any area of Medicine. In all, I am sure, dilemmas, problems and complaints resound with equal intensity. Since it is a specific event, we focused the discussion on the current wave of incorporation and the use of circulatory support devices. In fact, it is important to emphasize that the current methods for us have already been routine for other nations for decades. See the example of Extracorporeal membrane oxygenation (ECMO) (Fig. 1), incorporated into clinical practice about 40 years ago and still gets in our country the stamp of "experimental technique". This label is not given exclusively by the health insurance companies or even by the National Health System, with its well-known and alleged financial needs. We were appalled, when the Federal Council of Medicine gives a six-line opinion without any biographical source that gives them protection, saying that is an experimental procedure. On the other hand, we note that, despite this, the ANVISA (Brazilian Health Surveillance Agency) and the ANS (National Regulatory Agency for Private Health Insurance and Plans), respectively, authorized the incorporation of this technology in the country and put on the list of approved medical procedures for clinical use. The international and even national guidelines, some of them still "in process", recognize and define the criteria for recommendations and management of such equipment. Even then, the Federal Council of Medicine still considers it an "experimental technique".

Backed up by the medical knowledge built and assimilated over decades, I, as a doctor, am able to recommend and perform a widely supported procedure, but I may be accused of doing tests on human guinea pigs through the eyes of those who interpret and guard the Code of Medical Ethics. What to do? Should I give up on everything or run the risk of staining my unblemished career? Many of us are faced with this same conflict and fear.

More than a simple drama of consciousness, this dilemma ends up favoring those who, for financial reasons, hamper the development of the honorable "art of healing". I do not want to belittle or relegate the costs of incorporating these and other technologies. It is worth remembering that the final costs involving such equipments, most of the times, are higher when compared to the costs practiced in Europe and the USA. Do not tell me that this is due exclusively to the exchange-taxes binomial. We all have witnessed that the action of some bad professionals contribute to the price increase and non-viability of these technologies. However, supported in alleged technical opinions, the right to life is denied to those individuals who could take advantage of this primary legislation.

On the other hand, hospitals that try to put themselves at the forefront of this technological incorporation collide with successive negative responses from the institutions, and frequently, succumb to reality and avoid the quixotic confrontation. After all, many other legitimate interests are at stake.

In a scenario where the characters involved in the health system do not get along, that it is, the most fragile one that would be the direct beneficiary of new technologies - the patient, needs the aid of justice. The big problem is that justice does not understand anything about the subject and is put in the condition to arbitrate disputes and demands that never stop coming. How can someone without the minimum technical and scientific insight decide on such delicate and risky issues? Well, we delegate the guidelines and decisions to lay people. It happens because we are incapable of talking it over and jointly define the criteria for these incorporating technologies occur. But who should have the initiative to start the dialogue and reconciliation? Medical associations, health institutions or the government? It does not matter. If the willingness for dialogue, common sense and the interest to do something new do not permeate the behavior of these characters, nothing will happen. We could give the government the role to lead this great discussion, something that has never happened before. Of course we could, but the immobility of the Brazilian state at this time prevents simple and elementary actions from being put into action.

In broad terms, this was the synthesis of a nearly two-hour debate, which maintained the high level of information and respect. A debate that deserves and needs to be held in other scientific meetings as a way to clarify and especially raise awareness that medical science itself is not enough. It is supported especially in social science, where the contradictory factors and reconciliations are the mainspring for the scientific update, the constant search for quality of care we want, can and must provide with respect, knowledge and maturity.

**Helmgton Souza - MD, PhD, UNIFESP-EPM,** Coordinator of the Advanced
Heart Failure and Circulatory Assistance Center of Hospital Brasília and Hospital
Coração do Brasil - Distrito Federal, Program Director of 456^th^ ECMO
Center. Brasília, DF, Brazil.

